# Demographics, risk factors associated with malignant progression, and pregnancy outcomes of hydatidiform mole: A retrospective cohort study in Shanghai, China

**DOI:** 10.3389/fonc.2025.1643590

**Published:** 2025-07-30

**Authors:** Tingting Zhu, Guohua Zhu, Jiahui Ma, Xiaojuan Yu, Tingting Chen, Xiang Tao, Li Sun, Xin Lu, Yan Du

**Affiliations:** ^1^ Department of Gynecology Oncology, Obstetrics and Gynecology Hospital of Fudan University, Shanghai, China; ^2^ Shanghai Key Lab of Reproduction and Development, Shanghai, China; ^3^ Shanghai Key Lab of Female Reproductive Endocrine Related Diseases, Shanghai, China; ^4^ Clinical Research Unit, Obstetrics and Gynecology Hospital of Fudan University, Shanghai, China; ^5^ Department of Pathology, Obstetrics and Gynecology Hospital of Fudan University, Shanghai, China; ^6^ Department of Ultrasound, Obstetrics and Gynecology Hospital of Fudan University, Shanghai, China

**Keywords:** hydatidiform mole, demographics, postmolar gestational trophoblastic neoplasia, risk factor, pregnancy outcome

## Abstract

**Objective:**

This study aimed to clarify the demographics, evaluate risk factors associated with malignant progression, and assess pregnancy outcomes among patients with hydatidiform mole (HM) using a large, retrospective cohort study in Shanghai.

**Methods:**

This retrospective cohort study included patients with pathologically confirmed HM from 2019 to 2023. Descriptive analyses were performed to describe the demographic characteristics, progression to postmolar gestational trophoblastic neoplasia (pGTN), and reproductive outcomes of patients. Univariate and multivariate logistic regression analyses were conducted to evaluate risk factors and develop predictive models for pGTN.

**Results:**

Of 506 patients with HM, the average age and gestational age at diagnosis were approximately 33 years and 10 weeks, respectively. During follow-up, 42 patients (8.3%) progressed to pGTN, all achieved complete response after treatment. Univariate and multivariate analyses revealed that significant risk factors for progression to pGTN included pathological type and maximum diameter of lesions by ultrasound pre-evacuation (*p* < 0.05 for both). A predictive model incorporating age, β-hCG ratio (before/after evacuation), and ultrasound characteristics (uterine/lesion ratio) demonstrated optimal performance and goodness of fit. Among the 304 patients who intended to conceive, 254 had documented reproductive outcomes (follow-up rate: 83.6%). Of these, 163 patients (64.2%) achieved successful re-pregnancy, including 131 (80.4%) livebirths.

**Conclusion:**

Our study provides a comprehensive update on the demographics, risk factors associated with progression to pGTN, and reproductive outcomes of patients with HM in Shanghai. The clinical application of the predictive model needs to be further verified in a longitudinal setting.

## 1 Introduction

Hydatidiform mole (HM) is a pregnancy-related condition predominantly affecting women of reproductive age, with potential for malignant progression (1). Global epidemiological patterns demonstrate marked geographical variation (2–5). Developed regions such as Europe and North America report comparatively low incidence rates of 1-3 cases per 1,000 pregnancies (or deliveries) (2–4), while historically, developing regions across Asia, Africa, and parts of South America have documented rates as high as 10 cases per 1,000 deliveries (5). However, emerging data suggest epidemiological convergence, with Asian incidence rates now comparable to Western countries (6). Specifically, recent reports from South Korea (1.1/1,000 pregnancies) (7) and Japan (1.02-2.70/1,000 live births) (8) reflect this epidemiological transition.

China’s HM epidemiology remains incompletely characterized, with only two nationwide studies conducted to date (9, 10). The initial 1979-1983 survey across 26 provinces, municipalities, and autonomous regions reported an incidence rate of 0.81/1,000 pregnancies (9), while a subsequent 1991–2000 hospital-based study across 7 provinces documented 2.50 cases/1,000 pregnancies (10). Since then, research on HM in China has primarily been regional and has shown disparities (11). In addition, China currently lacks a national GTD registry. Given the rapid demographic changes in China over the past few decades, the current incidence and temporal trends of HM remain largely unknown. Therefore, comprehensive characterization of HM demographics, risk factors, and outcomes is essential for optimal health resource allocation.

The preferred treatment for HM is evacuation, with most patients achieving spontaneous remission following the removal of molar tissue. However, there remains a risk of malignant progression to postmolar gestational trophoblastic neoplasia (pGTN) (1). HM can be classified into complete hydatidiform mole (CHM) and partial hydatidiform mole (PHM), which demonstrate significant differences in morphology, histopathology, cytogenetics, and risk of malignant progression (1, 12). The progression rates to pGTN are 15%-20% for CHM and 0.5%-5% for PHM, respectively (13, 14). Various clinical, genetic, and molecular factors have been identified as risk factors for pGTN. Well-studied risk factors include maternal age over 40 years, advanced gestational age, theca lutein cysts larger than 6 cm, uterine size exceeding gestational age, history of molar pregnancy, and pre-evacuation beta human chorionic gonadotropin (β-hCG) levels higher than 100,000 mIU/mL (15–19). Nevertheless, some controversies persist regarding these risk factors. From a genetic perspective, *NLRP7* mutations have been implicated in increasing the risks of both HM recurrence and pGTN development (20).

Current standard monitoring strategies for patients with HM after evacuation involve routine follow-up, which mainly relies on clinical variables, including regular measurement of serum β-hCG levels and imaging examinations such as ultrasound (5, 21). While these methods are effective for early detection and timely treatment of pGTN, frequent follow-up visits and repeated blood sampling can reduce patient compliance and impose significant psychological and economic burdens. Recent studies have shown that patients with GTD generally experience a lower quality of life after treatment compared to the general population, particularly in mental health, social functioning, and intention to conceive (22). Therefore, early identification of high-risk HM patients and the development of individualized management plans are crucial to optimizing outcomes.

This study aims to analyze the demographics, risk factors, and pregnancy outcomes of patients with HM, using data from the largest GTD treatment center in Shanghai, eastern China.

## 2 Materials and methods

### 2.1 Study design and participants

This large-scale retrospective cohort study was conducted from January 2019 to December 2023 at the GTD Treatment Center of the Obstetrics and Gynecology Hospital of Fudan University in Shanghai, China. Our center serves over 80% of the GTD patients in metropolitan Shanghai, which has a population of approximately 24.8 million. Inclusion criteria were: (1) histopathologically confirmed HM following evacuation at our hospital; (2) availability of complete clinical and pathological records; (3) absence of myometrial infiltration or metastasis; (4) documented β-hCG normalization for ≥6 months post evacuation, or subsequent diagnosis of pGTN; and (5) provided informed consent. Exclusion criteria were: (1) undetermined last menstrual period that precluding gestational age estimation; (2) incomplete clinical information; (3) loss to follow-up; and (4) concurrent malignancies or autoimmune diseases. The manuscript was prepared in accordance with the STROBE guidelines (23).

### 2.2 Variables and assessments

The following information was extracted from patient medical records: (1) demographic data (age, height, weight); (2) reproductive history (gravidity, parity, history of pathological pregnancies); (3) medical and family histories; (4) clinical presentations (vaginal bleeding, celialgia, hyperemesis gravidarum, signs of preeclampsia, hyperthyroidism); (5) β-hCG values before and after evacuation; (6) ultrasonographic findings; (7) histopathological classification (CHM or PHM).

Postoperative β-hCG levels were monitored every 1–2 weeks. While patients with PHM could discontinue follow-up after β-hCG normalization, those with CHM – given their higher risk of malignant progression - require monthly monitoring for 6 months following three consecutive normal β-hCG measurements.

The primary outcome was progression to pGTN from CHM, PHM, and/or unclassified HM. pGTN was diagnosed according to the International Federation of Gynecology and Obstetrics (FIGO) 2018 criteria (24). After excluding new pregnancies, pGTN was diagnosed if one of the following conditions was met: (1) serum β-hCG plateau (± 10%) across four measurements over ≥ 3 weeks (e.g., days 1, 7, 14, 21); (2) ≥10% β-hCG elevation in three consecutive weekly measurements (e.g., days 1, 7, 14); (3) histopathological diagnosis of invasive mole or choriocarcinoma. Time to malignant progression, type of tumor, FIGO stage and score at diagnosis, and chemotherapy regimens were recorded and analyzed.

Another main outcome of this study was pregnancy outcomes, including livebirths, expectants, miscarriages, and ectopic pregnancies.

### 2.3 Statistical analysis

Categorical variables were expressed as percentages (%), and compared using the Fisher’s exact test. The Shapiro–Wilk test was used to assessed whether continuous variables followed a normal distribution. Continuous data that conformed to a normal distribution were expressed by mean ± standard deviation (SD), and compared using Student’s t-test; variables that did not follow a normal distribution were presented as median (range), and compared using the Mann-Whitney U test.

Two parameters were calculated: the β-hCG ratio (β-hCG level pre-evacuation/first β-hCG level after evacuation within 48 hours) and the ultrasound ratio (maximum diameter of uterine/lesions by ultrasound pre-evacuation). β-hCG levels were categorized as >100,000 *vs.* ≤100,000 IU/L. Univariate and multivariate logistic regression models with estimated odds ratios (ORs) and 95% confidence intervals (CIs) were utilized to evaluate predictive factors of pGTN occurrence. Variable selection was primarily based on a comprehensive literature review, as well as clinical experience. Multivariate analysis was performed to account for potential confounders and imbalanced covariates. Variables with *p* < 0.05 in the univariate analysis or those with clinical significance were included in the multivariate analysis.

Different predictive models were constructed, and receiver operating characteristic (ROC) analysis was used to evaluate their predictive value. The area under the ROC curve (AUC) quantifies a model’s discriminatory ability, with values closer to 1.0 indicating better performance and generalization. The Akaike information criterion (AIC) and Bayesian information criterion (BIC) were calculated to assess the goodness of fit, with lower values indicating a better fit.

Fertility status was assessed using the following indicators: (1) re-pregnancy rate = (number of HM patients who conceived post-recovery / total number of patients attempting conception) ×100%; (2) livebirth rate = (numbers of livebirths / number of re-pregnancies) ×100%. The curves of re-pregnancy rates were assessed using the Kaplan-Meier method and log-rank test.

Missing data were excluded from the analyses, and all key assumptions underlying these analyses were verified. Statistical analyses were performed using SPSS 24.0 and R (version 4.1.2). A two-sided *p* < 0.05 denoted as statistical significance.

### 2.4 Ethics statement

Different databases were linked using the unique patient ID number. Once the dataset was constructed, all identifiable data were removed to protect patient’s privacy. This study has been approved by the Ethics Committee of the Obstetrics and Gynecology Hospital of Fudan University (No.: FCKIRB-2023-135). This study was conducted in accordance with the guidelines of the 2000 Declaration of Helsinki. Written informed consent was obtained from all women.

## 3 Results

**Figure 1:**
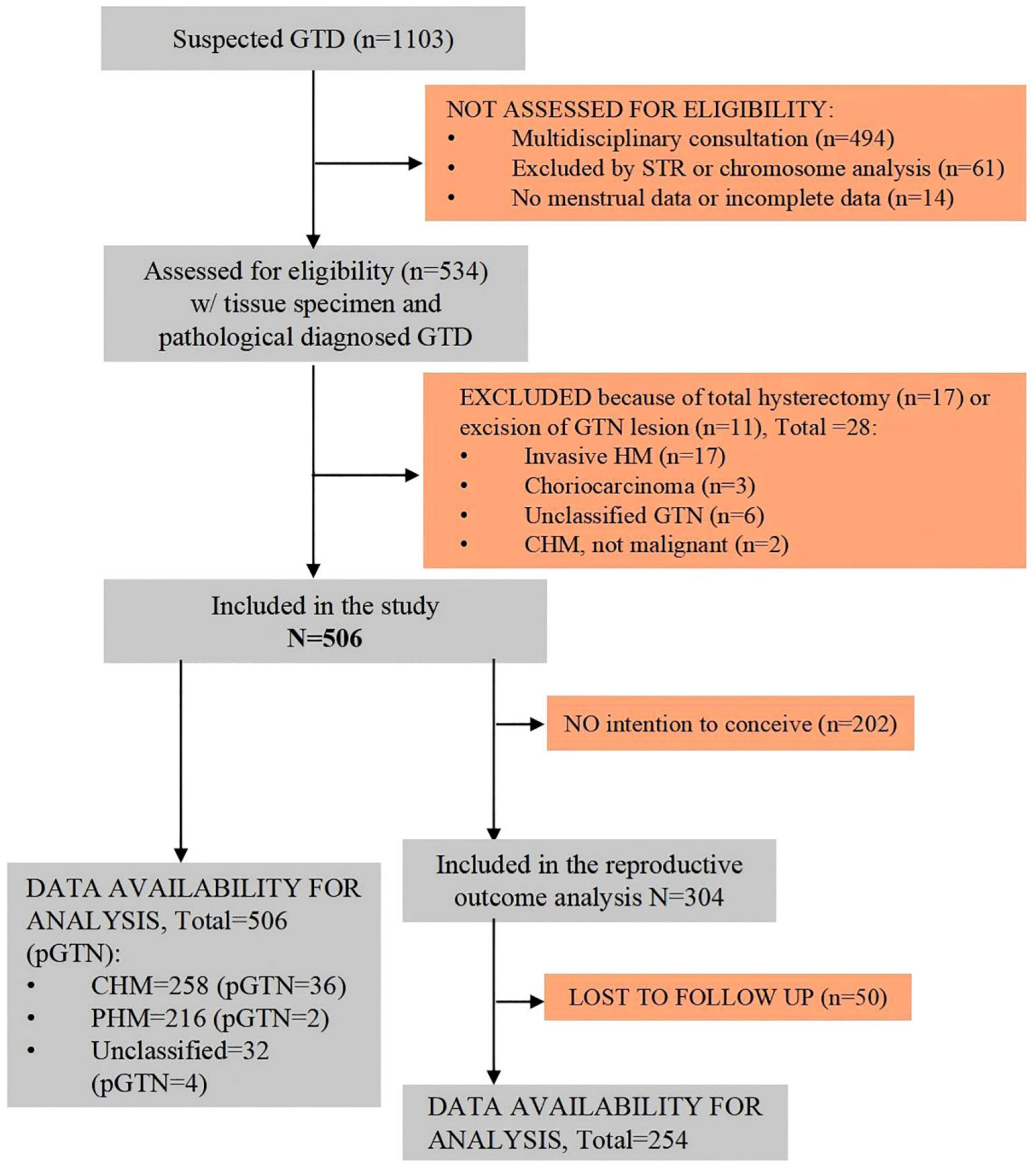
Flowchart of the study population. CHM, complete hydatidiform mole; GTD, gestational trophoblastic disease; GTN, gestational trophoblastic neoplasia; HM, hydatidiform mole; PHM, partial hydatidiform mole; pGTN, postmolar gestational trophoblastic neoplasia.

During the study period, there were a total of 1,103 suspected GTD cases at our GTD referral center. After excluding cases based on multidisciplinary consultation only (n=494), exclusion by short tandem repeats (STR) or chromosome analysis (n=61), and incomplete information (n=14), eventually 534 patients with tissue specimens were diagnosed with GTD by histopathology and/or short tandem repeat (STR) analysis at our referral center. Of these, 506 patients underwent uterine evacuation and were pathologically diagnosed with HM (**Figure 1**).

### 3.1 Demographics of participants

The average age of the 506 patients was 32.92 ± 7.48 years, and the average gestational age at diagnosis was 10.03 ± 3.60 weeks. The majority of the patients conceived naturally. The most common clinical presentation was vaginal bleeding (n=263, 52.0%), while a few patients reported celialgia and hyperemesis gravidarum. Approximately 42% of the patients were asymptomatic and were detected solely through ultrasound. Of all patients, 175 (34.6%) were initially diagnosed with HM, whereas more than half (n=293, 57.9%) were reported as missed abortion. Pathological diagnosis revealed that nearly half of the patients had CHM (n=258, 51.0%), while 216 (42.7%) had PHM. Additionally, 32 patients (6.3%) had unclassified HM and did not undergo STR analysis to determine their pathological type (**Table 1**). Secondary evacuation was performed in 30 patients, primarily due to residual tissue detected during follow-up. Additionally, 10 patients (aged 48–56 years) underwent total hysterectomy, including 1 emergency surgery for hemorrhage, and 2 cases following chemotherapy for malignancy, and 7 prophylactic resections. 

**Table 1:** Characteristics of HM and pGTN from year 2019–2023 at the GTD Center of OBGYN hospital in Shanghai, China.

	HM (n=506)	Spontaneous remission (n=464)	pGTN (n=42)	*p*
Age (year)	32.92 ± 7.48	32.59 ± 7.11	36.60 ± 10.13	0.001
Gravidity, median (range)	1 (0–12)	1 (0–12)	2 (0–6)	0.019
Parity, median (range)	0 (0–4)	0 (0–4)	1 (0–2)	0.054
Abortion history				0.053
No	243 (48.0%)	229 (49.4%)	14 (33.3%)	
Yes	263 (52.0%)	235 (50.6%)	28 (66.7%)	
Method of contraception				0.063
No	428 (84.6%)	398 (85.8%)	30 (71.4%)	
Condom	66 (13.0%)	55 (11.9%)	11 (26.2%)	
Contraceptive vaginal ring	11 (2.2%)	10 (2.2%)	1 (2.4%)	
Contraceptive pills	1 (0.2%)	1 (0.2%)	0 (0)	
History of infertility, n=505				0.242
No	483 (95.6%)	441 (95.2%)	100 (100%)	
Yes	22 (4.4%)	22 (4.8%)	0 (0)	
Mode of conception				0.657
Natural	484 (95.7%)	443 (95.5%)	41 (97.6%)	
ART^#^	13 (2.6%)	12 (2.6%)	1 (2.4%)	
Ovulation induction	9 (1.8%)	9 (1.9%)	0 (0)	
Gestational week	10.03 ± 3.60	9.93 ± 3.11	11.11 ± 7.04	**0.041**
Initial diagnosis				<0.001
HM	175 (34.6%)	141 (30.4%)	34 (81.0%)	
Missed abortion	293 (57.9%)	285 (61.4%)	8 (19.0%)	
Early miscarriage	21 (4.2%)	21 (4.5%)	0 (0)	
Ectopic pregnancy	12 (2.4%)	12 (2.6%)	0 (0)	
Other	5 (1.0%)	5 (1.1%)	0 (0)	
Pathological type				<0.001
CHM	258 (51.0%)	222 (47.8%)	36 (85.7%)	
PHM	216 (42.7%)	214 (46.1%)	2 (4.8%)	
Unclassified HM	32 (6.3%)	28 (6.0%)	4 (9.5%)	
P57, n=365				<0.001
Negative	231 (63.3%)	204 (61.3%)	27 (84.4%)	
Positive	122 (33.4%)	120 (36.0%)	2 (6.3%)	
Heterogenous	12 (3.3%)	9 (2.7%)	3 (9.4%)	
β-hCG pre-evacuation, IU/L	n=432, 103000.50 (47288.25-232192.25)	n=393, 86259.00 (40831.00-21244.00)	n=39, 249952.00 (108292.00-268400.00)	<0.001*
First β-hCG after evacuation, IU/L	n=457, 1196.36 (149.78-23304.50)	n=416, 629.81 (129.84-17577.25)	n=41, 20955.00 (5892.00-95760.50)	<0.001*
Days of β-hCG return to normal level	/	n=356, 55.04 ± 26.61	/	/
Average time to pGTN	/	/	N=42, 42.81 ± 27.05	/
Vaginal bleeding	263 (52.0%)	236 (50.9%)	27 (64.3%)	0.108
Celialgia	72 (14.2%)	68 (14.7%)	4 (9.5%)	0.490
Hyperemesis gravidarum	23 (4.5%)	22 (4.7%)	1 (2.4%)	0.711
Preeclampsia	2 (0.4%)	2 (0.4%)	0 (0)	1.000
Hyperthyroidism	3 (0.6%)	2 (0.4%)	1 (2.4%)	0.229
Ultrasonic appearance only	213 (42.1%)	200 (43.1%)	13 (31.0%)	0.144
Maximum diameter of uterine by ultrasound pre-evacuation (mm)	n=484, 74.21 ± 17.45	n=451, 73.29 ± 16.04	n=33, 86.76 ± 28.40	**0.011**
Maximum diameter of lesions by ultrasound pre-evacuation (mm)	n=487, 45.08 ± 24.25	n=452, 42.80 ± 21.47	n=35, 74.54 ± 36.49	**<0.001**
Second evacuation				0.002
No	476 (94.1%)	442 (95.3%)	34 (81.0%)	
Yes	30 (5.9%)	22 (4.7%)	8 (19.0%)	
Hysterectomy				<0.001
No	496 (98.0%)	461 (99.4%)	35 (83.3%)	
Yes	10 (2.0%)	3 (0.6%)	7 (16.7%)	

*Mann-Whitney U test.

^#^A patient underwent ART without a documented history of infertility.

ART, assisted reproductive technology; CHM, complete hydatidiform mole; GTD, gestational trophoblastic disease; β-hCG, beta human chorionic gonadotropin; HM, hydatidiform mole; pGTN, post-molar gestational trophoblastic neoplasia; OBGYN, Obstetrics and Gynecology Hospital of Fudan University; PHM, partial hydatidiform mole. bold values indicating *p*<0.05.

### 3.2 Malignant progression to pGTN

During the follow-up after evacuation, 464 patients with HM experienced spontaneous remission, while 42 (8.3%) progressed to pGTN. The malignant progression rate was 14.0% (36/258) for CHM and 0.9% (2/216) for PHM, respectively. The mean duration until β-hCG level normalization was 55.04 ± 26.61 days for the spontaneous remission group (n=356); while the mean duration to pGTN was 42.81 ± 27.05 days. Among the pGTN patients, five were diagnosed with invasive hydatidiform moles through histopathological evaluation, while the remaining patients lacked a definitive pathological classification. According to the FIGO prognostic risk scoring system, approximately 60% of the patients were classified as Stage III. Moreover, the majority of patients (n=35, 83.3%) were categorized as low-risk GTN (FIGO score <6) (**Table 2**).

**Table 2:** Characteristics of postmolar gestational trophoblastic neoplasia (pGTN).

	pGTN (n=42)
Age (Year)	
< 25	1 (2.4%)
25-29	17 (40.5%)
30-34	4 (9.5%)
35-40	5 (11.9%)
40-45	3 (7.1%)
< 45	12 (28.6%)
Mole type	
CHM	36 (85.7%)
PHM	2 (4.8%)
Unclassified HM	4 (9.5%)
Days to pGTN	42.81 ± 27.05
Pathological type	
Unclear	37 (88.1%)
Invasive hydatidiform mole	5 (11.9%)
Stage	
I	16 (38.1%)
II	1 (2.4%)
III	25 (59.5%)
FIGO score, median (range)	2.5 (0-8)
Low risk (score 0-6)	35 (83.3%)
High risk (score ≥7)	7 (16.7%)
First-line treatment	
Single-agent chemotherapy	32 (76.2%)
MTX	24
KSM	8
Multi-agent chemotherapy	10 (23.8%)
EMA/CO	8
EMA/EP	1
MTX+KSM	1
Second-Line treatment	
Single-agent chemotherapy: KSM	1 (2.4%)
Multi-agent chemotherapy: EMA/CO	4 (9.5%)
None	37 (88.1%)

CHM, complete hydatidiform mole; EMA/CO, etoposide, methotrexate, dactinomycin/cyclophosphamide, vincristine; EMA/EP, etoposide, methotrexate, dactinomycin/etoposide, cisplatin; FIGO, International Federation of Gynecology and Obstetrics; HM, hydatidiform mole; KSM, dactinomycin; MTX, methotrexate; pGTN, post-molar gestational trophoblastic neoplasia; PHM, partial hydatidiform mole. bold values indicating *p*<0.05.

In our cohort, the first-line treatment for pGTN predominantly involved single-agent chemotherapy, with more than three-quarters of patients receiving monotherapy with either methotrexate or dactinomycin. Less than one-quarter of the patients required combination therapy, primarily using the etoposide, methotrexate, dactinomycin, cyclophosphamide, and vincristine (EMA/CO) regimen (**Table 2**). Notably, 88% of patients achieved remission without any alterations to their chemotherapy regimen. After treatment, all pGTN patients were followed up regularly and remained relapse-free.

### 3.3 Risk factors associated with HM malignant progression

The results of the univariate logistic regression analysis indicated that several variables were significantly associated with the development of pGTN: age (OR=1.06, 95% CI: 1.03-1.11), pathological type (CHM *vs.* PHM: OR=17.35, 95% CI: 4.13-72.96), pre-evacuation β-hCG level (>100,000 *vs.* ≤100,000 IU/L: OR=4.27, 95% CI: 1.91-9.52), first β-hCG level after evacuation (>100,000 *vs.* ≤100,000 IU/L: OR=3.62, 95% CI: 1.64-8.02), maximum diameter of the uterus (OR=1.03, 95% CI: 1.02-1.05) and maximum diameter of the lesions (OR=1.04, 95% CI: 1.03-1.05) measured by ultrasound before evacuation. Additionally, two calculated indicators were significantly associated with pGTN development: the β-hCG ratio (OR=0.56, 95% CI: 0.32-0.96) and the ultrasound index (OR=0.03, 95% CI: 0.01-0.14) (**Table 3**).

**Table 3:** Univariate and multivariate logistic regression analysis results.

Variable	Univariate analysis		Multivariate analysis	
	OR (95% CI)	*p*	OR (95% CI)	*p*
Age	1.06 (1.03-1.11)	**0.001**	0.97 (0.92-1.03)	0.287
Gravidity	1.17 (0.98-1.39)	0.094	/	/
Gestational week	1.06 (1.00-1.13)	0.058	/	/
CHM vs. PHM (n=474)	17.35 (4.13-72.96)	**<0.001**	17.61 (2.25-137.55)	**0.006**
β-hCG pre-evacuation (>100,000 vs. ≤100,000 IU/L) (n=432)	4.27 (1.91-9.52)	**<0.001**	1.00 (1.00-1.00)	0.530
First β-hCG after evacuation (>100,000 vs. ≤100,000 IU/L) (n=457)	3.62 (1.64-8.02)	**0.001**	1.00 (1.00-1.00)	0.468
β-hCG pre-evacuation/First β-hCG after evacuation	0.56 (0.32-0.96)	**0.035**	/	/
Maximum diameter of uterine by ultrasound pre-evacuation (mm, within one week) (n=484)	1.03 (1.02-1.05)	**<0.001**	1.01 (0.99-1.04)	0.383
Maximum diameter of intrauterine space-occupying lesions by ultrasound pre-evacuation (mm) (n=487)	1.04 (1.03-1.05)	**<0.001**	1.04 (1.02-1.06)	**0.001**
Maximum diameter of uterine by ultrasound pre-evacuation/Maximum diameter of intrauterine space-occupying lesions by ultrasound pre-evacuation)	0.03 (0.01-0.14)	**<0.001**	/	/

Adjusted for age, pathologic type (CHM vs. PHM), β-hCG pre-evacuation, first β-hCG after evacuation, menstruation status after treatment.

95% CI, 95% confidence interval; β-hCG, beta human chorionic gonadotropin; CHM, complete hydatidiform mole; OR, odds ratio; PHM, partial hydatidiform mole. bold values indicating *p*<0.05.

Multivariate analysis revealed that only the type of mole (CHM *vs.* PHM: OR=17.61, 95% CI: 2.25-137.55) and the maximum diameter of the lesions measured by ultrasound before evacuation (OR=1.04, 95% CI: 1.02-1.06) remained significant risk factors of progression to pGTN (**Table 3**).

### 3.4 Predictive model of HM malignant progression

**Figure 2:**
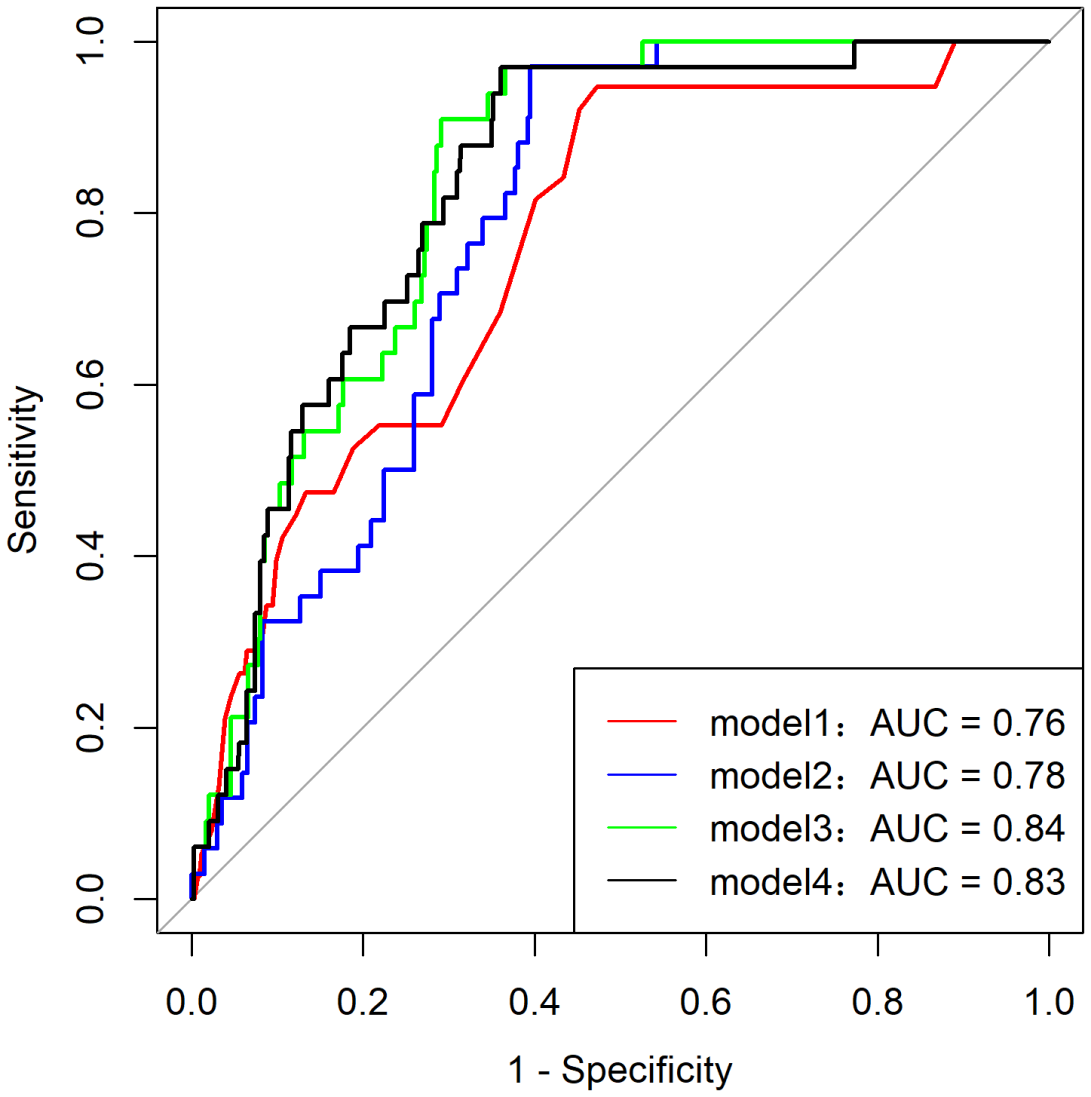
Area under the ROC curve (AUC) of Model 1-4.

Among the 216 patients with PHM, only two developed pGTN during follow-up, rendering the multivariate model that included the HM subtype unstable. Additionally, the HM subtype showed a strong correlation with ultrasound indices (*p* for correlation <0.001). Consequently, various predictive models of pGTN were developed and compared (**Figure 2**). Among these models, Model 3, which incorporated age, β-hCG ratio, and ultrasound index, achieved the highest AUC of 0.84 (95% CI: 0.78-0.89, *p* < 0.001, sensitivity=0.91 and specificity=0.71). Furthermore, Model 3 also had the lowest AIC (180.93) and BIC (196.72) values, indicating the best goodness of fit (**Table 4**).

**Table 4:** Different predictive models of HM progression to pGTN.

Variable	Model 1	Model 2	Model 3	Model 4
	OR (95% CI), *p*	OR (95% CI), *p*	OR (95% CI), *p*	OR (95% CI), *p*
Age	1.05 (1.01-1.08), **0.018**	1.02 (0.98-1.06), 0.288	1.02 (0.98-1.06), 0.406	1.03 (0.99-1.08), 0.152
CHM vs. PHM (n=474)	16.13 (3.82-68.09), **<0.001**	31.42 (2.86-344.94), **0.005**		
β-hCG pre-curettage/first β-hCG after curettage		0.63 (0.42- 0.95), **0.027**	0.53 (0.33-0.84), **0.007**	
Maximum diameter of uterine/lesions by ultrasound pre-curettage			0.05 (0.01-0.21), **<0.001**	0.04 (0.01-0.16), **<0.001**
AUC (95% CI), *P*	0.76 (0.69-0.83), <0.001	0.78 (0.72-0.84), <0.001	0.84 (0.78-0.89), <0.001	0.83 (0.77-0.89), <0.001
Sensitivity and Specificity	0.95 and 0.53	0.97 and 0.60	0.91 and 0.71	0.97 and 0.64
AIC	231.84	190.84	180.93	200.25
BIC	244.32	206.52	196.72	212.78

95% CI, 95% confidence; AIC, Akaike information criterion; AUC, area under the ROC curve; β-hCG, beta human chorionic gonadotropin; BIC, Bayseian information criterion; CHM, complete hydatidiform molar; HM, hydatidiform molar; OR, odds ratio; pGTN, post-molar gestational trophoblastic neoplasia; PHM, partial hydatidiform molar. bold values indicating *p*<0.05.

### 3.5 Pregnancy outcomes of patient with HM

**Figure 3:**
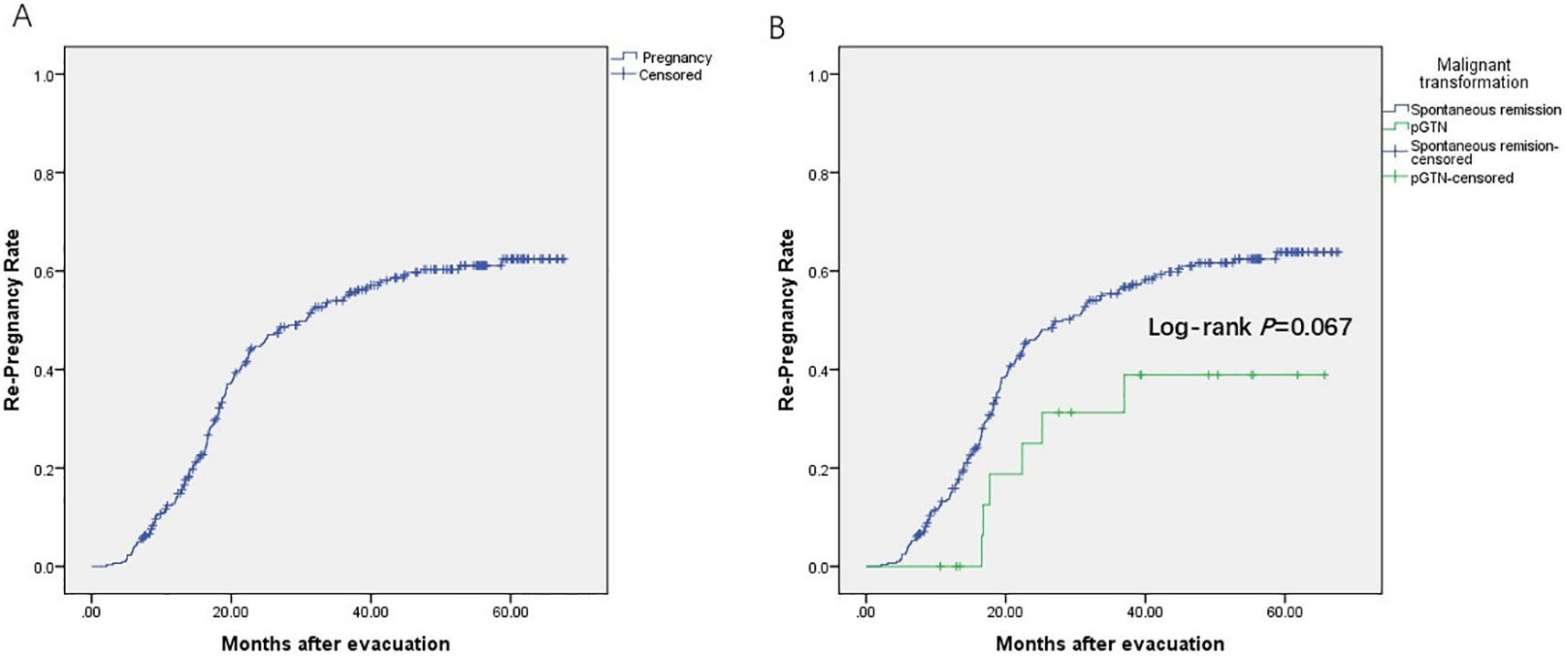
Re-pregnancy rate. **(A)** Patients with fertility intention and follow-up information (n=254); **(B)** Spontaneous remission group (n=285) *vs.* pGTN group (n=19), including loss of follow-up (censored).

Among the 506 patients with HM, 304 (60.1%) intended to conceive. As of July 19, 2024, among those with fertility intention and follow-up information (n=254, follow-up rate: 254/304 = 83.6%), 163 achieved successful re-pregnancy (re-pregnancy rate: 163/254 = 64.2%) (**Figure 3A**), and 131 had livebirths (livebirth rate: 131/163 = 80.4%). Additionally, 9 were expectants, 21 experienced miscarriages (13 early miscarriages and 8 with unspecified timing), and 2 had ectopic pregnancies. The ongoing pregnancy rate was 85.9% (140/163). There was no significant difference in the re-pregnancy rate between the spontaneous remission group and the pGTN group (*p* = 0.067, **Figure 3B**). Specifically, among the 17 patients with pregnancy intentions in the pGTN group, 5 achieved subsequent pregnancies and successfully delivered.

## 4 Discussion

In this large retrospective cohort study, we described the demographics of patients with HM from 2019 to 2023. We validated that HM subtypes (CHM *vs.* PHM) are an independent risk factor for malignant progression in HM. Additionally, we identified ultrasound measurements as potential effective predictors for monitoring progression to pGTN. We subsequently developed a predictive model for pGTN risk, which may prove useful in the clinical management of patients with HM. Furthermore, we reported that pregnancy outcomes in these patients were comparable to those in the general population, suggesting that a history of HM or subsequent pGTN does not adversely affect fertility.

Previous studies have demonstrated that the clinical presentation of HM has evolved with advancements in first-trimester ultrasonography and β-hCG measurement. The average gestational age at diagnosis has decreased from 16 weeks to approximately 9 weeks. This shift has led to a decline in the incidence of presenting classic symptoms, such as vaginal bleeding, anemia, preeclampsia, excessive uterine enlargement, and hyperemesis gravidarum, along with an increase in asymptomatic patients (25, 26). A study conducted at the New England Trophoblastic Disease Center between 1994 and 2013, which included 180 patients with CHM, reported that the incidence of vaginal bleeding was significantly lower than in the preceding decade (46% *vs.* 84%, *p* < 0.001). However, vaginal bleeding remained the most prevalent symptom, noted in 80 patients (27). Consistent with these findings, our data demonstrated that the average gestational age at diagnosis was approximately 10 weeks, as ultrasound examinations are routinely conducted in the first trimester. The predominant clinical presentation was vaginal bleeding, which accounted for 52% of patients, followed by asymptomatic individuals, who comprised 42% of the cohort. Only a small proportion exhibited abdominal pain and hyperemesis gravidarum. Additionally, we observed a reduction in typical symptoms and an increase in asymptomatic patients, with more than half (57.9%) of the patients initially diagnosed with a missed abortion. This underlines the necessity for improved diagnostic protocols to differentiate HM from other miscarriages, particularly in the context of atypical histopathological findings.

However, early diagnosis has not significantly reduced the risk of malignant progression, which persists at around 10% (8, 27). The pathologic type of HM has been consistently identified as a significant predictor of pGTN in both our analysis and previous studies (13), highlighting the critical importance of accurate subtype classification for effective clinical management. In our cohort, the ratio of CHM to PHM (258/216) was lower than that reported in a northern Chinese cohort (413/263) (11), which may explain the relatively lower malignant progression rate observed in our study population. Overall, our findings revealed a malignant progression rate of approximately 8%, which is consistent with previous studies (8, 11). Previous research has indicated that pGTN is usually diagnosed several weeks to months after molar evacuation (5). In our cohort, the mean time to malignant progression was approximately 6 weeks following evacuation. Nevertheless, the early diagnosis of HM poses significant challenges due to its atypical histopathological features, which may resemble edematous villi or trisomies. Specific analytical techniques such as p57KIP2 immunostaining are increasingly employed in the diagnosis of CHM. As most CHM cases lack maternal chromosomes, they typically exhibit negative p57KIP2 immunostaining, as this marker is normally maternally expressed. In contrast, both PHM and non-molar abortions contain maternal chromosomes, resulting in positive p57KIP2 immunostaining, which can complicate pathological differentiation. While the latest World Health Organization (WHO) blue book has recommended STR analysis for definitive PHM diagnosis, its clinical implementation remains constrained by substantial costs and specialized personnel requirements (28).

Serum β-hCG levels, reflecting trophoblast proliferation activity, are an important indicator for monitoring the prognosis of HM patients after treatment. Current clinical guidelines recommend monitoring β-hCG levels every 1–2 weeks after evacuation. For cases of PHM, follow-up can be discontinued upon β-hCG normalization, whereas CHM patients require extended monitoring with monthly β-hCG testing for 6 months after achieving three consecutive negative results (29). However, frequent blood sampling during follow-up may impose physical discomfort, psychological distress, and financial burdens on patients, potentially compromising patient compliance. This not only hinders effective monitoring and management of patients but also affects their overall well-being. Notably, recent data from the UK national retrospective population study suggest that surveillance protocols could be safely modified to one confirmatory normal hCG value for CHM patients achieving β-hCG normalization within 56 days of evacuation (30).

Our study identified that ultrasound characteristics as valuable predictors of pGTN. Transvaginal ultrasound, a non-invasive and cost-effective approach, is widely employed in diagnosing and monitoring HM. Previous studies have suggested that increased myometrial vascularization and lower uterine artery Doppler indices may be related to invasive diseases (31, 32). However, only a few studies have characterized the morphology and vascular patterns of early GTN lesions (33, 34). It was reported that about 80% (28/36) of pGTN patients presented with detectable uterine lesions at the time of diagnosis (34), suggesting that patients with HM who do not meet the β-hCG diagnostic criteria but exhibit suspicious lesions in the myometrial layer on ultrasound should undergo prompt evaluation for potential malignant progression. It is likely that ultrasound examinations may reveal uterine lesions or vascular flow alterations precede measurable changes in serum β-hCG levels. Consequently, patients with suspected ultrasound characteristics are possibly at high risk and warrant vigilant follow-up.

In contrast to previous prediction models relying solely on β-hCG values (35–37), our study developed comprehensive predictive models incorporating ultrasound parameters and multiple clinical characteristics to assess malignant progression risk. Through systematic evaluation of various prediction models, we identified Model 3 with age, hCG ratio, and ultrasound indices, as the most effective. Model 3 achieved superior discriminative ability, as evidenced by the highest AUC value among all tested models (AUC=0.84, 95% CI: 0.78-0.89). This robust performance reflects the model’s multidimensional design that combines clinical, biochemical, and imaging parameters, thereby capturing the complex pathophysiology of pGTN development. The model’s enhanced reliability was further supported by its favorable information criteria values (AIC=180.93; BIC=196.72), indicating an optimal balance between model parsimony and predictive accuracy. Notably, the significant correlation between ultrasound indices and HM subtypes provides biological plausibility for including imaging parameters in the prediction algorithm. The incorporation of ultrasound metrics is particularly clinically relevant given their non-invasive nature, widespread availability, and ability to provide real-time morphological assessment. When combined with serial β-hCG monitoring, these imaging parameters offer valuable complementary information for early risk stratification. While Model 3 shows promising predictive accuracy and clinical utility for identifying high-risk pGTN cases, external validation in independent cohorts is warranted to confirm its generalizability. Future studies should investigate its potential for guiding personalized clinical management decisions and optimizing patient outcomes.

Our current livebirth rate was comparatively lower than rates reported in previous studies (38). This discrepancy may be attributable to the fact that most of our patients diagnosed and treated with fertility intentions in 2023 (n=35) have not yet achieved successful conception (n=32, 91.4%). As our longitudinal follow-up data mature, we anticipate the fertility outcomes will improve. A meta-analysis of six studies including a total of 25,222 patients found that adverse obstetric outcomes in patients with molar pregnancies were comparable to those in the general population (38). Although HM does not appear to adversely affect future pregnancy outcomes, our findings revealed that only 60% (304/506) of patients expressed an intention to conceive. This underscores the critical need for psychological assessment and targeted interventions, such as preconception counseling, to alleviate their fears and hesitations. In the context of China’s rapid socioeconomic development, particularly in the more affluent eastern coastal regions, we observed distinct demographic shifts including advanced maternal age. The mean age of our cohort was close to 33 years, slightly older than the 31.7 ± 6.8 years reported from northern China (11). Additionally, an increasing proportion of women are resorting to assisted reproductive technology (ART). While ART utilization in our cohort remained relatively low (n=13, 2.6%), its potential influence on gestation-related diseases merits further investigation.

To the best of our knowledge, our study provides a comprehensive analysis of the demographics and pregnancy outcomes of patients with HM in eastern China, utilizing a large hospital-based retrospective cohort. Our study advances previous research by systematically evaluating multiple traditional risk factors using our large dataset. Previous studies have predominantly focused on individual risk factors and/or had small sample sizes, making it challenging to draw robust conclusions. Importantly, we developed a predictive model that integrates several simple predictive variables, which are effective in screening for high-risk patients. The implementation of this model could optimize clinical practice by reducing unnecessary interventions and improve patient outcomes. Our study can provide evidence for more efficient health resource allocation, risk stratification, and development of personalized clinical management and follow-up strategies for patients with HM.

However, as this study is a retrospective, single-center analysis, it carries inherent limitations, including potential selection and/or recall biases. Our hospital serves as one of China’s largest referral centers for GTD. Notably, patients referred to our center tend to have higher educational attainment and socioeconomic status compared to the general population. Consequently, these particularities may limit the generalizability of our findings, necessitating external validation of the proposed predictive model. To address this, a prospective, multi-center cohort study is currently being conducted. This follow-up study aims to validate our preliminary results, provide updated epidemiological on HM in China, and contribute to evidence-based strategies for enhancing population health in general and women’s health in particular.

In conclusion, in our large hospital-based cohort study of over 500 patients in eastern China, we reported demographics and pregnancy outcomes of HM, which were similar to those observed in developed regions and countries. The identified risk factors and the predictive model we developed offer valuable tools for risk stratification and the clinical management optimization. While the clinical benefits of these approaches require further validation, our findings provide updated evidence to inform both clinical practice and healthcare policy formulation regarding HM management.

## Data Availability

The data analyzed in this study is subject to the following licenses/restrictions: the datasets analyzed during the current study are available upon request from the principal investigator (Xin Lu, Email: xin_lu@fudan.edu.cn) and meeting the criteria of Ministry of Science and Technology of the People’s Republic of China. Requests to access these datasets should be directed to Dr. Xin Lu, Email: xin_lu@fudan.edu.cn.
